# Adaptive divergence in body size overrides the effects of plasticity across natural habitats in the brown trout

**DOI:** 10.1002/ece3.579

**Published:** 2013-05-23

**Authors:** Björn Rogell, Johan Dannewitz, Stefan Palm, Jonas Dahl, Erik Petersson, Anssi Laurila

**Affiliations:** 1 School of Biological Sciences/Monash UniversityClayton, 3100, Melbourne, Australia; 2 Animal Ecology/Department of Ecology and Evolution, Evolutionary Biology CenterNorbyvägen 18D, 752 36, Uppsala, Sweden; 3 Institute of Freshwater Research, Department of Aquatic Resources, Swedish University of Agricultural SciencesStångholmsvägen 2, 178 93, Drottningholm, Sweden; 4 Kristianstads Vattenrike, Biosphere ReserveSpannmålsgatan 11, Kvarnen, Kristianstads kommun, 291 80, Kristianstad, Sweden

**Keywords:** Genetics, growth, local adaptation, *Salmo trutta*, survival

## Abstract

The evolution of life-history traits is characterized by trade-offs between different selection pressures, as well as plasticity across environmental conditions. Yet, studies on local adaptation are often performed under artificial conditions, leaving two issues unexplored: (i) how consistent are laboratory inferred local adaptations under natural conditions and (ii) how much phenotypic variation is attributed to phenotypic plasticity and to adaptive evolution, respectively, across environmental conditions? We reared fish from six locally adapted (domesticated and wild) populations of anadromous brown trout (*Salmo trutta*) in one semi-natural and three natural streams and recorded a key life-history trait (body size at the end of first growth season). We found that population-specific reaction norms were close to parallel across different streams and *Q*_ST_ was similar – and larger than *F*_ST_ – within all streams, indicating a consistency of local adaptation in body size across natural environments. The amount of variation explained by population origin exceeded the variation across stream environments, indicating that genetic effects derived from adaptive processes have a stronger effect on phenotypic variation than plasticity induced by environmental conditions. These results suggest that plasticity does not “swamp” the phenotypic variation, and that selection may thus be efficient in generating genetic change.

## Introduction

It is well established that antagonistic genotype by environment interactions for fitness can create adaptive spatial variation, where different genotypes are favored under different environmental conditions (Merila and Crnokrak 2001; Kawecki and Ebert 2004; Leinonen et al. 2008, 2013). Since the potential of populations to adapt to specific conditions carries strong implications for nature conservation and our understanding of evolutionary patterns, the quantification of local adaptation has become an important task in biological research.

Local adaptation may, however, be inferred in several ways, one of the most widely used being reciprocal translocation studies, where different populations are reared in a reciprocal manner in their locations of origin (Hereford 2009). Antagonistic patterns in the performance of different populations across environments and, especially, populations performing best in their native environment are inferred to reflect local adaptation (Kawecki and Ebert 2004; Hereford 2009). Although an efficient method for studying local adaptation, translocation studies suffer from two logistic shortcomings. First, it is often difficult to rear organisms originating from multiple populations under natural “common garden” conditions. This is especially the case in freely moving organisms. Second, since the objective is to examine antagonistic patterns of fitness of different genotypes across environments, it is crucial to have a correct measure of fitness. As fitness is a composite trait, its correct estimation is not a simple task (Shaw et al. 2008). Indeed, traits closely linked to fitness, such as survival or reproductive success, are likely to be involved in trade-offs with other fitness-related traits (De Jong and Van Noordwijk 1992; Reznick et al. 2000). Consequently, the inference of local adaptation is highly dependent on how fitness is estimated.

The above complications have led to the development of alternative ways to examine presence of local adaptation based on comparing observed genetic divergence in traits of interest to a scenario of neutral divergence. One such a methodology is *Q*_ST_*–F*_ST_ comparison (Spitze 1993), where neutral genetic divergence as estimated from neutral molecular markers (*F*_ST_) is compared to the divergence in a quantitative trait (*Q*_ST_). *Q*_ST_ is estimated as follows:


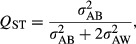


where σ^2^_AB_ is the additive genetic variance component explained by population differences, whereas σ^2^_AW_ is the within-population genetic variation of the trait. If *Q*_ST_ << *F*_ST_, stabilizing selection is inferred, if *Q*_ST_ >> *F*_ST_ divergent selection is inferred, and if *Q*_ST_ and *F*_ST_ are of the same magnitude, the trait under study is assumed to have evolved in a neutral manner.

In order to obtain reliable estimates of genetic variances for populations, it is crucial that the traits are recorded under common conditions, that is, when the populations compared are exposed to the same environment, since environmental effects may inflate variation in trait divergence (Pujol et al. 2008; Alho et al. 2010, 2011). In a similar vein, the estimation of genetic variances often requires controlled environmental conditions (Weigensberg and Roff 1996). Consequently, the vast majority of the studies using *Q*_ST_*–F*_ST_ comparisons to infer local adaptations have been conducted in laboratory conditions (Leinonen et al. 2008; Pujol et al. 2008). If the intention of the study is to infer local adaptation *as it would appear under natural conditions*, these preconditions make the strong assumption that there are no population × environment interactions in the focal trait. Although the inference for local adaptation via *Q*_ST_ can be influenced by variation in experimental conditions (e.g., Richter-Boix et al. 2010; Hangartner et al. 2012), few studies have examined the presence of local adaptation using both translocation experiments and *Q*_ST_*–F*_ST_ comparisons, where the assumption of no population × environment interactions can be tested. Such interactions are also important from an evolutionary perspective, as the speed at which an evolutionary response may precede is likely to be dependent on the amount of plasticity of the trait (Price et al. 2003). In cases where the genetic effects are overridden by plastic responses to different environments, plasticity may impede an evolutionary response (e.g., Price et al. 2003; West-Eberhard 2003), suggesting that plasticity may play a key role at early stages of divergence. However, the relative amount of variation in fitness traits explained by environmental versus genetic effects under natural conditions has remained an unexplored topic in evolutionary biology.

This question is especially relevant in managed systems like salmonid fish populations, where individuals from foreign populations have frequently been introduced into native populations. If genotypes of different origin are highly plastic, we may expect that there is only weak or no selection against nonadapted (foreign) genotypes. On the other hand, if phenotypic variation is mainly genetic, we may expect that natural selection is acting directly on genetic variance and that nonadapted genotypes will be selected against. Sea-run brown trout (*Salmo trutta*) is commonly managed by sea-ranching, where breeding fish are caught from the wild, the offspring are reared in a hatchery and subsequently released into the wild at the smolt stage, when juvenile salmonids migrate to the sea (e.g., Petersson et al. 1996). The hatchery conditions differ strongly from the species' natural habitats, and adaptive divergence, where hatchery populations of the brown trout have higher growth rates than wild populations has been demonstrated (e.g., Rogell et al. 2012). In addition, genetic selection gradients for larger body size were very steep for wild trout populations reared in hatchery environments, indicating that body size is strongly genetically correlated with a causal trait upon which hatchery selection is acting (Rogell et al., unpubl. ms.). This is not surprising, since growth rate and body size frequently vary across environmental clines. Body size is often closely associated with fitness, and a large body size generally incurs higher reproductive capacity and competitive ability (Arendt 1997; Dmitriew 2011). However, the fact that many populations express submaximal growth rates is generally explained by costs of high growth in terms of a higher sensitivity to predators and environmental stress (Arendt 1997; Dmitriew 2011). For example, adaptive divergence in intrinsic growth rates in amphibians has been found across gradients of latitudinal climatic variation (Palo et al. 2004) and environmental stress (Rogell et al. 2009; Hangartner et al. 2012). Thus, the fast-growing hatchery populations are likely to be more competitively dominant/aggressive, but they are also likely to exhibit a more risk-taking behavior, which makes them more exposed to predation in the wild (Biro et al. 2004; Sundström et al. 2004).

Here, we examine the consistency of local adaptation across a range of environmental conditions, as well as the amount of phenotypic variance explained by population and environment, in a large-scale experiment conducted during two successive years. In a previous study, we reared several sea-run brown trout populations over the first growth season under controlled hatchery conditions and in a semi-natural experimental stream, respectively, to assess within- and among population variation, and to estimate *Q*_ST_ for body size and survival (Rogell et al. 2012). Microsatellite markers were used to estimate neutral genetic differentiation (*F*_ST_) between the study populations and to determine population and family origin of the experimental fish (Rogell et al. 2012). In a parallel study reported in this article, all parental crosses from all populations were stocked into natural streams devoid of trout, located close to the native streams of the focal populations. This allowed us to compare trait values of different populations in a semi-reciprocal manner.

The objectives of this study were (i) to investigate if adaptive patterns of brown trout life-history traits are consistent across stream environments and (ii) to assess the importance of population origin and rearing environment on growth and survival. Since our previous results showed that reaction norms in trout body size and survival across hatchery and experimental stream conditions were parallel (Rogell et al. 2012), we expected population × rearing environment interactions on size to be rather small, which would indicate that adaptive patterns of brown trout life-history traits are consistent across stream environments. Predictions for survival differ, however, since a trade-off between growth rate and survival can be assumed. As captive hatchery conditions lack predators, we expected the hatchery populations to have a stronger competitive ability in the experimental stream free from predatory fish. On the other hand, as fish predators are present in the natural streams, we expected hatchery trout to be more adversely affected by predation than their wild conspecifics in those environments. As we have no prior expectations, due to lack of previous studies, of how much variation in life-history traits that are explained by adaptive divergence versus plastic processes we assessed the importance of population origin and rearing environments on body size. We note that a stronger genetic than plastic component would allow a more efficient selection against nonnative genotypes.

## Material and methods

Two experiments were performed in two successive years (Rogell et al. 2012). In both experiments, fish from different populations were caught from the wild, mated artificially, and the progeny of these fish were released into an experimental stream and natural streams similar to the native streams.

### Study populations

**Figure 1:**
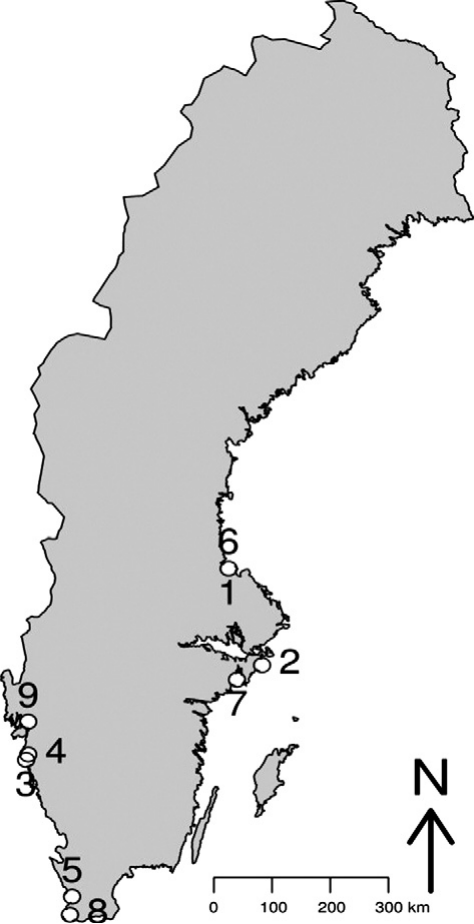
Map over Sweden showing the original locations of experimental populations: 1. Dalälven, 2. Åvaån, 3. Jörlandaån, 4. Norumsån, 5. Kävlingeån, and rearing environments: 6. Experimental stream, 7. Mölnboån, 8. Sularpsbäcken, 9. Brattåsbäcken.

In River Dalälven (60°38′N, 17°26′E) on the Swedish east coast (Fig. 1; mean discharge 350 m^3^/sec), large-scale releases of hatchery trout of local origin have been conducted for several decades. The trout are managed by sea-ranching. Presently, there are two independent hatchery lines from River Dalälven (genetically isolated for two vs. eight generations) that are kept separated by artificial markings (different fin-clipping). Åvaån (59°10′N, 18°22′E) is a small stream on the Swedish east coast (mean discharge 0.1 m^3^/sec, Fig. 1) with a wild sea trout population that for some decades has also been used for artificial propagation (see Rogell et al. 2012). The juvenile trout from Åvaån are kept in standard hatchery facilities for their first year, after which they are moved to net pens in the sea ca. 25 km from Åvaan (59°45′N, 18°15′E), where they are later released as smolts. The released trout exhibit a homing behavior to the net pens, and the production of stocking material is based on released hatchery origin fish of returning to the pens. This was also where our parental fish were caught and Åvaan is thus considered a hatchery population. Jörlandaån (57°59′N, 11°48′E) and Norumsån (58°03′N, 11°49′E) are small neighboring streams (mean discharge of 0.5 and 0.2 m^3^/sec, respectively) on the Swedish west coast (Fig. 1). Both streams harbor wild, productive sea-run brown trout populations with no documented releases of hatchery-reared trout. Kävlingeån (55°44′N, 12°60′E) in southernmost Sweden is a small river (mean discharge of 11 m^3^/sec), which harbors a native trout population that has most likely not been affected by releases of hatchery-reared trout (Eklöv 2000).

### Rearing environments

Due to genetic and other risks associated with releasing nonnative trout, the native streams (where the experimental parents were collected) could not be used as rearing environments. We therefore chose alternative streams so that each population had a closely located “home” stream with physical and biological characteristics as similar to the native stream as possible. The chosen streams fulfill today requirements for trout, and two of them (Sularpsbäcken and Brattåsbäcken, Fig. 1) have also harbored trout in the past. However, these streams presently lack trout populations because of man-made migration obstacles located further downstream.

For River Dalälven (Fig. 1), the experimental stream located at the research station in Älvkarleby was used as the experimental “home” stream. The experimental stream is 110 m long with a total area of 345 m^2^ and consists of four pools with riffles in between (for details see Dannewitz et al. 2003). A tube supplies the stream with river water from a nearby hydroelectric power dam in River Dalälven. The experimental stream was drained before the experiments so that no fish predators were present in the system. Fish released into the stream had access only to naturally occurring food and were exposed to some predation by American mink *Mustela vison* and gray heron *Ardea cinerea*.

Mölnboån (Fig. 1) was used as “home” river for Åvaån trout. The river is located on the Swedish east coast and has only secondary contact with the Baltic Sea. The stream has suitable spawning and nursery areas for trout, but because of limited migration possibilities the stream has probably not been inhabited by a trout population in the past.

Sularpsbäcken (Fig. 1) is a tributary to River Kävlingeån in southern Sweden. Sea trout was previously present, but construction of downstream migration barriers eradicated the original population. The water quality is excellent and the spawning and nursery areas are considered very suitable for trout.

Brattåsbäcken (Fig. 1) is situated close to the streams Norumsån and Jörlandaån. Also, this stream likely harbored sea trout in the past, but the population disappeared when the stream was culverted further downstream. However, the spawning and nursery areas upstream are still considered suitable for trout.

### Experiment 1

Experiment 1 was conducted in 2003 and involved five populations: Dalälven (2nd and 8th hatchery generation populations, additional information in Fig. 1), Åvaån, Jörlandaån, and Norumsån.

Mature fish were caught in their native rivers in autumn 2002. In Kävlingeån, Jörlandaån, and Norumsån, sexually mature adults were caught by electrofishing in the rivers, whereas Åvaån adults were caught with gill nets. In Dalälven, adult trout were caught in a permanent trap used for catching mature fish for the supportive breeding program. The trout were stripped in the field, and eggs and milt were transferred to the experimental facilities at the Swedish University of Agricultural Sciences' research station located near the mouth of River Dalälven (Älvkarleby municipality), where controlled crosses were performed.

In total, 12 males and 12 females from each of the two Dalälven populations and Norumsån, 11 males and 11 females from Åvaån, and 10 males and 10 females from Jörlandaån were used for controlled crosses between 22 October and 9 November. Each female was artificially mated with a unique male from the same population, resulting in a total of 12*3 + 11 + 10 = 57 full-sib families, which were incubated in family-specific trays in the hatchery.

On 29 March 2003, 200 eyed eggs from each family were transferred into the experimental stream. The number of eggs transferred to Mölnboån and Brattåsbäcken on the same date varied between 131 and 800 per family. The large variation in egg number among families reflected the large variation among females in body size and thus fecundity. For three families, the incubated eggs were too few to allow transfer into all streams. Therefore, only 54 families were stocked to Mölnboån and Brattåsbäcken. The total numbers of eggs stocked were 11,400 in the experimental stream and 32,803 in each of Mölnboån and Brattåsbäcken. In all streams, eyed-stage eggs were stocked in Vibert incubation boxes (Vibert 1949, 400–500 eggs per box); the eggs from all families were first pooled to avoid confounding effects of incubation site on subsequent performance.

The experimental stream was drained on 8 September 2002 and the young-of-the-year (YOY) trout were recovered by hand-netting. Mölnboån and Brattåsbäcken were electrofished on 2 and 9 September, respectively (each stream was fished during a single day). A total of 45, 128, and 739 offspring were caught in Mölnboån, Brattåsbäcken, and in the experimental stream, respectively. Body length of all fish was measured, and tissue samples were taken for parentage analyses.

### Experiment 2

Experiment 2 was conducted in 2004 and involved four populations: Dalälven (8th hatchery generation), Åvaån, Kävlingeån, and Jörlandaån. Mature fish were caught in autumn 2003. A total of 12 males and 12 females from each of the four populations (8th hatchery generation Dalälven, Åvaån, Kävlingeån, and Jörlandaån) were used for artificial crosses, done between 22 October and 24 November. We followed the same procedures as in Experiment 1; however, an incomplete diallel mating design was this time applied to allow separation of additive genetic variance from maternal and nonadditive effects (Lynch and Walsh 1998). Eggs of two dams were fertilized with milt from each of two sires to create six 2 × 2 breeding matrices for each population, except for Kävlingeån where only four complete 2 × 2 matrices and two additional (maternal) half-sib families could be produced, with eggs of two unique dams fertilized by each of two unique sires, creating four full-sib crosses. Hence, the total number of unique crosses (full-sib families) was 90 (24 from each population, except from Kävlingeån which had 18 unique crosses).

The same environments and procedures as in Experiment 1 were used, except that an additional stream (Sularpsbäcken) in the same drainage system as Kävlingeån (Fig. 1) was included to obtain a “home” stream also for this population. One hundred eyed eggs from each parental cross were stocked into the experimental stream. In Mölnboån, Brattåsbäcken, and Sularpsbäcken, the number of eggs stocked from each full-sib family varied between 17 and 400. The stockings were made on 25 and 26 March 2004. Five parental crosses that were stocked into the experimental stream were not introduced into the three natural streams because of too few eggs available (cf. experiment 1). The total number of eggs stocked was 9000 in the experimental stream, whereas it varied between 23,998 and 24,095 in Mölnboån, Brattåsbäcken, and Sularpsbäcken. We chose to introduce a larger number of eggs in the natural streams in order to compensate for presumably higher mortality rates in these environments.

Sampling of offspring took place between 14 and 16 September 2004, using the same methods as in Experiment 1. A total of 50, 34, 196, and 831 trout from Mölnboån, Brattåsbäcken, Sularpsbäcken, and the experimental stream were caught, respectively.

### Parentage assessment

Assessment of family and population origin of all juveniles caught after the first growth season in experiments 1 and 2 was performed using eleven highly variable microsatellite loci, as described in Rogell et al. (2012). Parentage was assessed by comparing the alleles at a given locus from each offspring with the alleles in each of the potential parental crosses using the software WHICHPARENTS (http://bml.ucdavis.edu/research/research-programs/conservation/salmon-research/salmon-genetics-software/). The used markers gave a high discriminatory power, and >99% of the sampled offspring could be assigned unambiguously to a single parental pair. Individuals that matched two or more parental crosses were excluded from the statistical analyses.

### Statistical analysis

#### Estimation of *Q*_ST_

With the exception of the experimental stream, the survival rates were too low for calculation of genetic variances as intended by the experimental design. Instead, we used the formula by Brommer (2011) where the *Q*_ST_-proxy *P*_ST_ is estimated as follows:


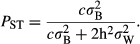


Here, σ^2^_B_ denotes the phenotypic variation between populations, *c* denotes the proportion of the total variance across populations presumed to be due to additive genetic effects, h^2^ is the narrow sense heritability, and σ^2^_W_ is the phenotypic variation within populations (residual variation).

In a vast majority of contemporary common garden experiments, similar to the present one, the factor *c* has been assumed to have a value of one (Leinonen et al. 2008; Pujol et al. 2009). When the heritability (h^2^) is unknown, different values for the heritability can be simulated to examine its effect on *Q*_ST._ However, since the experimental stream had rather low mortality, we could obtain an estimate of heritability in this specific rearing environment, we used this value and assumed equal variances across the other environments. Although genetic variances and heritabilities may differ across populations, this assumption is well in line with the equal additive genetic variance within populations used in *Q*_ST_ studies. It should be noted that *Q*_ST_ = *P*_ST_, when *c* = 1 and the additive genetic variances within populations are known (Brommer 2011). The heritability estimate was obtained from a Bayesian model containing the random effects population and family [Experiment 1] or sire and dam [Experiment 2]. The model was fit with the R package “MCMCglmm” as described below.

#### Variance explained by population and rearing environment

To explain sources of variation in body size, we calculated the intraclass correlation, which is a measure of the fraction of the total variance attributed to a particular factor. For this, we used linear mixed models where population origin and rearing stream were fit as random effects, and the grand mean was fit as the sole fixed effect. Intraclass correlations were thus calculated as follows:


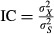
, where σ^*2*^_*X*_ is the specified variance component (population origin or rearing stream) and σ^*2*^_*S*_ is the total variation (the sum of all variance components, including the residual variance).

#### Variances

Variances and intraclass correlations were estimated using linear mixed models in a Bayesian setting. Specifically, the models were fit using a gibbs sampler implemented in the R package “MCMCglmm” (R Development Core Team 2009; Hadfield 2010). Population, stream and their interaction were included as fixed effects, and parental cross was included as a random effect. For each of the models, six parallel chains were run for two million iterations, from which 800,000 iterations were discarded as burn-in. After burn-in, every 800th iteration was sampled, yielding a total posterior sample size of 6×1500 = 9000 per model. Flat priors were used for the fixed effects, whereas locally uninformative priors were used for the random effects, both representing little prior knowledge. All auto-correlations were within the interval −0.1 and 0.1, and the 97.5% quantile of the Gelman–Rubin test statistic was below 1.2 in all cases, indicating only weak autocorrelations and that the models had converged. To further validate the results, the same linear mixed models were also fit using Restricted Maximum Likelihood; these models gave results highly congruent to the Bayesian models (results not shown).

#### Survival

The analysis of survival was complicated by the overall very low survival rates in the natural streams, as well as the uneven number and skewed distribution of the number of eggs introduced into these environments. To account for these complications, differences in survival were addressed using a parametric bootstrap approach, from which inferences were made based on the 95% bootstrap confidence intervals. Within each of the natural streams, we estimated the proportion survival within each family. From the resulting Binomial distributions describing the observed proportion of surviving versus dead individuals within each family, we made 1000 random draws, and on these pseudo-values we estimated the proportion of surviving individuals in order to obtain a parametric bootstrap distribution. To remove biases derived from the different number of stocked eggs, the size of the random draw was determined by cutoff values based on the distribution of the number of introduced eggs per family. These cutoff values (one per experiment) were chosen so that each family should have at least one surviving offspring from the expectations of the grand mean, under which it made little sense to calculate the number of surviving individuals. Families for which the cutoff value was higher than the number of introduced eggs (i.e., where the expected value was zero surviving individuals) were discarded from the survival analysis. The cutoff value was determined as 425 introduced eggs for Experiment 1 and 100 eggs for Experiment 2 (resulting in 27 and 63 excluded families, respectively). The Binomial sampling procedure was repeated 1000 times, and population mean survival was calculated from family means. More elaborate linear models with Binomial and Poisson errors (number of released eggs used as an offset variable) were also tested, but were found to not converge satisfactorily.

The experimental stream is very productive, lacks fish predation, and probably therefore had a substantially higher survival than the natural streams. When the experimental stream was included in the analysis, the results gave a significant interaction between rearing environment and population. However, this interaction was solely based on the difference in survival between the experimental stream and the natural streams, reflecting the general difference in mortality schemes between these environments. This means that the interaction was only informative of the survival probabilities in the experimental stream; a population with high survival in the experimental stream would have a bigger drop in survival to the natural streams than a population with low survival in the experimental stream. Consequently, and since population survival rates in the experimental stream have been published elsewhere (Rogell et al. 2012), we did not include the experimental stream in the survival analysis.

## Results

### Body length

**Figure 2:**
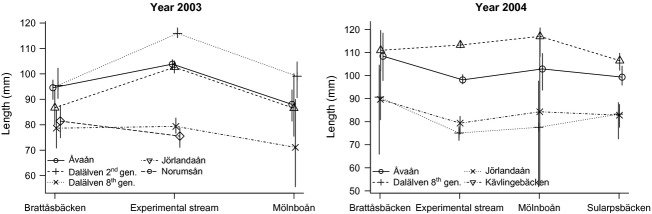
Reaction norms for estimated means (with 95% credibility intravals) of body length for the trout populations in years 2003 and 2004 across a total of four different rearing environments.

**Figure 3:**
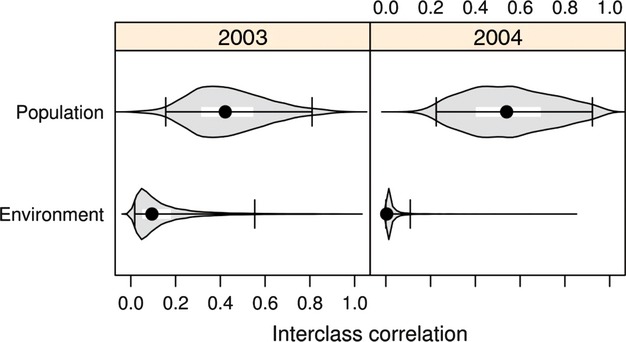
Violin plots depicting posterior distributions of intraclass correlation for population and environment in the two experiments. The dots indicate the median of the posterior probabilities and error bars its associated 95% quantiles.

**Figure 4:**
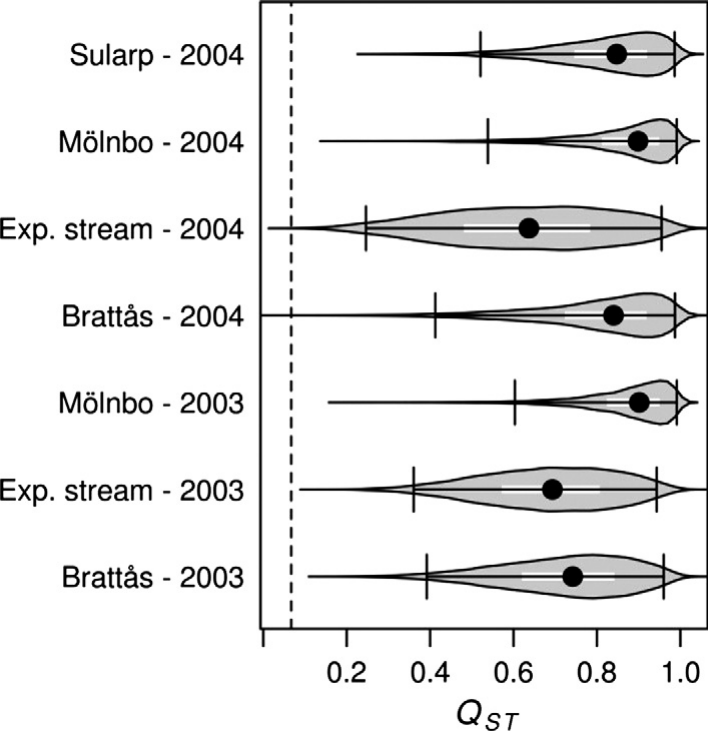
Violin plots illustrating *Q*_ST_ for body length among populations. Results are shown separately for different combinations of years and rearing environments. The dashed line represents the upper 95% quantile for *F*_ST_ (i.e., the neutral expectancy). The dots indicate the median of the posterior probabilities and error bars its associated 95% quantiles. Note that the median *Q*_ST_ overlap with the 95% quantiles in all cases.

Trout from the hatchery stocks were larger in all habitats in both years. The interactions between rearing environment and population origin were weak, with reaction norms being close to parallel (Fig. 2). When assessing the proportion of variation explained by population origin and stream environment, the intraclass correlation for population origin clearly exceeded that for stream in both experiments (Fig. 3). The relatively fixed divergence in body length among the populations across streams yielded large *Q*_ST_ values that were well beyond *F*_ST_ in all cases, and their magnitude was rather similar across rearing habitats and years (Fig. 4).

### Survival

As mentioned above, average survival differed greatly between the experimental and the natural streams. There was some variation in survival across the natural streams, with Sularpsbäcken showing overall higher survival than in the other streams. However, we found no significant differences in survival among populations within any of the natural streams (Fig. S1). Although the hatchery stocks had a higher survival in the experimental stream (Rogell et al. 2012), we could see no clear patterns or directionality regarding survival differences between populations in the natural streams.

## Discussion

We found that the evolutionary background of local brown trout populations explained a larger proportion of the phenotypic variation in an important life-history trait (body size) than plastic environmentally induced effects across natural rearing habitats. The small effect sizes of the population × environment interactions together with significant *Q*_ST_ values further suggest that the adaptive divergence in body size remains constant across a range of local stream environments. Given that the speed of an evolutionary response may be hampered by abundant plastic variation in the trait, our results also suggest that the selection on the phenotypic variance is likely to induce fast evolutionary responses (Price et al. 2003).

Theory predicts that populations can adapt locally when there are antagonistic patterns in fitness across different environmental conditions (Kawecki and Ebert 2004). Although we could not detect any significant differences in survival, our fitness component, *Q*_ST_ for body size was significantly higher than *F*_ST_ in all environments, and environment explained a smaller proportion of the phenotypic variation than population origin. Since *Q*_ST_ represents a test for local adaptation, the very weak interactions between population and rearing environment provide indirect evidence for antagonistic fitness patters for populations reared in different environments. As previously shown, these patterns are likely to be due to selection for higher growth rates in the hatchery-reared populations (Rogell et al. 2012; Rogell et al. unpubl. ms.). The overall higher growth rates in the hatchery populations can be due to increased competition and lack of predation mortality in the captive environment (e.g., Petersson et al. 1996). Higher growth rates are often associated with behaviors such as increased risk taking and may thus carry cost in terms of increased predation in natural habitats (Biro et al. 2004; Sundström et al. 2004). Previous studies have found that animals often decrease their foraging activity in environments with predators, which may translate into reduced growth rate (Lima 1998; Tollrian and Harvell 1999).

However, our results suggest that the fast growth rates of hatchery populations are likely to be expressed in environments both with and without fish predation. Since these genotypes will be fast growing across all environmental conditions, the divergence by hatchery rearing may carry costs in natural environments. Hatchery fish have been shown to have lower fitness under natural conditions compared to wild fish. For example, Araki et al. (2007, 2009) showed that hybrids between hatchery and wild steel head trout suffered a ∼40% reduction in fitness compared to wild fish under natural conditions. Such fitness losses are likely to reflect adaptive genetic changes associated with hatchery rearing (Araki et al. 2007; Araki et al. 2009; Rogell et al. 2012), but are these fitness losses likely to be constant across natural environments? We here show that the adaptive patterns of body size induced by hatchery rearing are similar across several natural habitats. Our results thus indicate that the phenotypic variation is to a large extent determined by genetic rather than environmental factors, and that hatchery adaptations may be efficiently selected against in the natural environments.

The methodology to infer selection based on *Q*_ST_*–F*_ST_ comparisons suffers from several methodological complications. Studies on the precision of *Q*_ST_ estimates have shown that the variance of *Q*_ST_ estimates is high and that there is a tendency for a downward bias, particularly when the number of populations is low (<20) and true *Q*_ST_ is high (O'Hara and Merilä 2005). As this would suggest that our *Q*_ST_ estimates are conservative, we do not find this a major drawback. Considering the low number of populations and streams in this study, Bayesian models have the advantage that model uncertainty is correctly summarized, and thus that correct confidence intervals for the parameter estimates can be constructed (Berger 1985). An additional source of error is the putative presence of maternal effects in the estimated among- and (broad sense) within-population variance components. Although we cannot exclude the potential confounding role of maternal effects, the rather similar results across the 2 years suggest that maternal effects were of minor importance in this study. Finally, we cannot exclude the possibility that our results are affected by competitive interactions as the hatchery fish are likely to be stronger competitors compared to the wild fish.

While our attempts to estimate survival in the natural streams were hampered by the generally low recapture rate in these streams, survival was considerably higher in the experimental stream (Rogell et al. 2012). The higher survival observed in the experimental stream could be due to the high productivity of this stream in combination with a lower predation pressure. Also, trout released in the experimental stream could have been less inclined to leave the stream because of the trap situated in the downstream part, whereas trout released in the natural streams (devoid of trout) could disperse and utilize larger areas of the streams (Elliot 1994; Rodriguez 2002), which in combination with predation may have resulted in lower recapture rates. However, previous studies have found that juvenile trout that are out-competed at their original areas suffer high mortality rates (Elliot 1986), and we thus argue that recapture rate may still be a relevant proxy for natural survival in the natural environments. As increased predation mortality is expected to be one of the main costs of high growth rates, we expected the fast-growing populations hatchery populations (from Dalälven and Åvaån) to express lower survival in the natural streams. No such pattern could be detected, and we were not able to make any inferences on population differences in survival due to the low recapture rates that gives very low statistical power.

Previous studies on genetic and phenotypic variation in salmonid fishes have found adaptive among-population variation in growth (e.g., Lahti et al. 2001; Fraser et al. 2011; Rogell et al. 2012), as well as plastic variation in relation to environmental factors like temperature and the amount of resources (Brett 1979; Klemetsen et al. 2003). Our results show that growth across different stream environments was quite similar, suggesting – perhaps surprisingly – a comparably low importance of local conditions affecting growth. The low plasticity found within the populations suggests that environmental variation is unlikely to buffer against natural selection, and that removal of maladapted genotypes may be fast under certain conditions.

On the other hand, it has been shown that adaptive divergence within species may affect ecological interactions and thus community structure (Bassar et al. 2010, 2012). It is thus not unlikely that the ecological niche may differ between hatchery and wild trout, and our results suggest that such differences may remain constant over a range of environmental conditions. Indeed, both conservation and fisheries biology harbor a great interest on organisms' potential to adapt to varying environmental conditions. For example, harvest, or its collateral effects, may change a species' evolutionary dynamics in a mode antagonistic to natural selection (e.g., Olsen et al. 2004), possibly resulting in decreased fitness of the harvested species and changes in community structure. Our results suggest that hatchery selection may have a stronger effect on key life-history parameters than environmental conditions, emphasizing the possibility that hatchery selection may also affect ecological interactions in stream environments following release or escape of domesticated populations.

To summarize, we have found that reaction norms for body size during the first growth season were rather parallel across several natural streams, and that population origin explained a larger part of the variation in growth than plastic effects induced by different environmental conditions. This, in turn, indicates that the relative performance is similar across a range of natural environments, and that higher growth rates induced by adaptation to hatchery conditions may be persistent across natural environments. According to life-history theory, a higher growth rate should occur at costs of other fitness components. In the present case, plasticity did not swamp the genetic differences, suggesting that fitness costs mediated by selection may be very strong. Such costs are likely to affect fitness of hatchery-reared fish in the wild. However, due to very low survival rates, we could not properly evaluate the effect of rearing environment on survival in the natural streams. Finally, it should be stressed that population differences (or a lack of them) seen during early life stages does not necessarily correlate well with lifetime success in survival and reproduction (cf. McGinnity et al. 2003), and further studies linking juvenile performance and adult fitness are thus needed.

## Supporting Information

**Figure S1.** Average survival rates (with 95% confidence intervals) for compared trout populations within combinations of natural rearing environments (streams) and experimental years. None of the five population comparisons yielded a statistically significant difference in survival.
