# Supplementary material for: Adaptive divergence in body size overrides the effects of plasticity across natural habitats in the brown trout
Source: Ecol Evol. 2013 May 23;3(7):1931–41. doi: 10.1002/ece3.579 (PMC3728935; doi:10.1002/ece3.579)
Supplement: Supplementary file 1 [file ece30003-1931-SD1.pdf]

Supplementary information

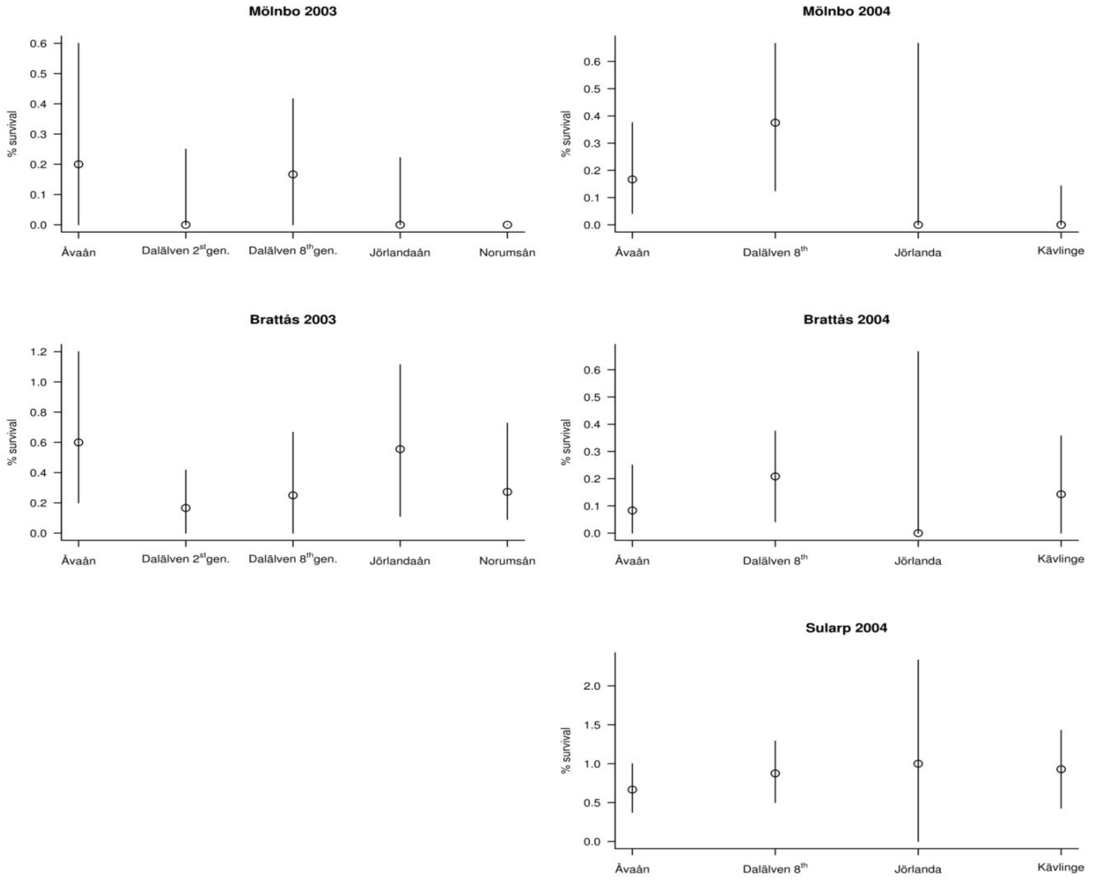

Supplementary figure 1. Average survival rates (with 95% confidence intervals) for compared trout populations within combinations of natural rearing environments (streams) and experimental years. None of the five population comparisons yielded a statistically significant difference in survival.
